# Correction: Second-shell modulation on porphyrin-like Pt single atom catalysts for boosting oxygen reduction reaction

**DOI:** 10.1039/d4sc90219j

**Published:** 2024-11-06

**Authors:** Tayyaba Najam, Syed Shoaib Ahmad Shah, Hanqing Yin, Xin Xiao, Shamraiz Talib, Qianqian Ji, Yonggui Deng, Muhammad Sufyan Javed, Jie Hu, Ruo Zhao, Aijun Du, Xingke Cai, Qiang Xu

**Affiliations:** a Institute for Advanced Study, Shenzhen University Shenzhen 518060 China cai.xingke@szu.edu.cn; b College of Physics and Optoelectronic Engineering, Shenzhen University Shenzhen 518060 China; c Department of Chemistry, School of Natural Sciences, National University of Sciences and Technology Islamabad 44000 Pakistan; d QUT Centre for Materials Science, Queensland University of Technology (QUT) 2 George Street Brisbane 4000 Australia; e Shenzhen Key Laboratory of Micro/Nano-Porous Functional Materials (SKLPM), SUSTech-Kyoto University Advanced Energy Materials Joint Innovation Laboratory (SKAEM-JIL), Department of Chemistry and Department of Materials Science and Engineering, Southern University of Science and Technology (SUSTech) Shenzhen 518055 China xuq@sustech.edu.cn; f Advanced Materials Chemistry Centre (AMCC), SAN Campus, Khalifa University Abu Dhabi P. O. Box 127788 United Arab Emirates; g College of Mechatronics and Control Engineering, Shenzhen University Shenzhen 518060 PR China; h Institute of Carbon Neutrality, Zhejiang Wanli University Ningbo 315100 China; i Institute for Integrated Cell-Material Sciences (WPI-iCeMS), Kyoto University Yoshida, Sakyo-ku Kyoto 606-8501 Japan

## Abstract

Correction for ‘Second-shell modulation on porphyrin-like Pt single atom catalysts for boosting oxygen reduction reaction’ by Tayyaba Najam *et al.*, *Chem. Sci.*, 2024, https://doi.org/10.1039/d4sc03369h.

The authors regret that one of the affiliations (affiliation *h*) was incorrectly shown in the original manuscript. The corrected list of affiliations is as shown here.

The Royal Society of Chemistry apologises for these errors and any consequent inconvenience to authors and readers.

